# Bronchoscopic Diagnosis of Severe Respiratory Infections

**DOI:** 10.3390/jcm13196020

**Published:** 2024-10-09

**Authors:** Maire Röder, Anthony Yong Kheng Cordero Ng, Andrew Conway Morris

**Affiliations:** 1 School of Clinical Medicine, Addenbrooke’s Hospital, University of Cambridge, Hills Road, Cambridge CB2 0QQ, UK; meb85@cam.ac.uk; 2 Department of Medicine, Addenbrooke’s Hospital, University of Cambridge, Hills Road, Cambridge CB2 0QQ, UK; aykn2@cam.ac.uk; 3 Division of Immunology, Department of Pathology, University of Cambridge, Tennis Court Road, Cambridge CB2 0QQ, UK; 4 JVF Intensive Care Unit, Addenbrooke’s Hospital, Hills Road, Cambridge CB2 0QQ, UK

**Keywords:** bronchoscopy, intensive care, respiratory infections, pneumonia

## Abstract

The diagnosis of severe respiratory infections in intensive care remains an area of uncertainty and involves a complex balancing of risks and benefits. Due to the frequent colonisation of the lower respiratory tract in mechanically ventilated patients, there is an ever-present possibility of microbiological samples being contaminated by bystander organisms. This, coupled with the frequency of alveolar infiltrates arising from sterile insults, risks over-treatment and antimicrobial-associated harm. The use of bronchoscopic sampling to obtain protected lower respiratory samples has long been advocated to overcome this problem. The use of bronchoscopy further enables accurate cytological assessment of the alveolar space and direct inspection of the proximal airways for signs of fungal infection or alternative pathologies. With a growing range of molecular techniques, including those based on nucleic acid amplification and even alveolar visualisation and direct bacterial detection, the potential for bronchoscopy is increasing concomitantly. Despite this, there remain concerns regarding the safety of the technique and its benefits versus less invasive sampling techniques. These discussions are reflected in the lack of consensus among international guidelines on the topic. This review will consider the benefits and challenges of diagnostic bronchoscopy in the context of severe respiratory infection.

## 1. Introduction

Respiratory failure is the most common reason for admission to the intensive care unit (ICU), and identifying its cause is critical for effective management. It is important to differentiate between sterile and infective causes of lung inflammation, and in infective causes, identification of the causative organism helps inform rational antimicrobial therapy. Common differentials for acute respiratory failure are infectious pneumonia, sterile direct lung injury, and acute respiratory distress syndrome (ARDS) arising from extrapulmonary insults.

Globally, the most common cause of respiratory failure requiring ICU admission is pneumonia. Despite its importance, there remains a paucity of evidence-based management of this condition [1]. Pneumonia is also the most common cause of ICU-acquired secondary infection [2]. The distinct categories of pneumonia encountered in the ICU include community-acquired (CAP), hospital-acquired (HAP), and ventilator-associated (VAP) pneumonia. These types of pneumonia differ in their microbial precipitants, and to some extent, the host responses directed against them.

Due to its ubiquity, patients with acute respiratory failure are commonly assumed to have pneumonia and frequently receive empiric antimicrobial therapy. When this therapy is either inappropriate (i.e., does not target the organisms present) or unnecessary (i.e., attempts to treat sterile lung injury), patients may come to harm [3,4]. This also has a negative impact on antimicrobial stewardship and efforts to limit antimicrobial resistance [5].

Invasive respiratory sampling for the diagnosis of severe pneumonia remains an area of active discussion and disagreement in critical care practice. Whether its risks are outweighed by its benefits has not yet been sufficiently systematically examined for a clear consensus to be reached. Furthermore, the impact of the growing suite of optical and molecular diagnostic techniques that can be used alongside bronchoscopy remains to be evaluated. Currently, there is significant heterogeneity in the diagnostic approaches employed by clinicians globally [6], and clinical guidelines are vague or divergent [7,8].

Although international guidelines discuss diagnostic management, their implementation is inconsistent [6] and the evidence underlying these recommendations is relatively sparse. A major decision in the diagnostic process in cases of suspected pneumonia is the method by which lower respiratory tract samples are obtained. Here, we describe the current practice of bronchoscopy and non-invasive respiratory sampling in the ICU; review the existing evidence concerning the utility of bronchoscopy in severe respiratory infections; then reflect on this in the context of current guidelines, highlight areas of uncertainty, and propose directions for future study.

### 1.1. Definition, Epidemiology, and Importance of Pneumonia

Pneumonia is defined as an inflammatory alveolar infiltrate and is normally triggered by an infectious agent, most commonly of bacterial origin. The global incidence of pneumonia is around 360 million cases per year [9], with a case fatality rate of 0.66%, which rises by at least an order of magnitude in severe cases [10]. It is therefore a leading global cause of death due to infection; in 2016, it was the primary cause of over 2 million deaths worldwide, around 650,000 of which were in children under five years of age. Nearly 5% of all deaths worldwide are due to pneumonia, and the majority of these are bacterial or viral in aetiology, with modest contributions from fungal and parasitic pathogens.

Respiratory viruses and *Streptococcus pneumoniae* are common causes of CAP, whilst nosocomial infections such as HAP and VAP are more commonly caused by Gram-negative bacteria and *Staphylococcus aureus*. A significant challenge in the diagnosis of pneumonia is that microbial cultures are frequently negative and a causal pathogen is only identified in approximately one-third of patients [2]. Additionally, many bacteria that cause pneumonia are also commonly found in the upper respiratory tract as commensal organisms [11].

In ventilated patients, the proximal lower respiratory tract (e.g., the trachea) is similarly rapidly colonised by these commensal organisms [12]. The clinician seeking a microbial diagnosis is therefore faced with the dual dilemmas of missing causal organisms and detection of commensals. This, coupled with the wide range of sterile causes of pulmonary inflammation, illustrates the challenge of diagnosing acute inflammatory respiratory failure.

Consensus criteria to grade the severity of community-acquired and nosocomial respiratory infections remain elusive, partly due to the breadth of causal pathogens, pathophysiologic endotypes of pulmonary infection [5], and international heterogeneity in management. The consensus guidelines for severe CAP [13] and HAP/VAP [14] agreed between the European Respiratory Society, European Society of Intensive Care Medicine, and the European Society of Clinical Microbiology and Infectious Diseases and Latin American Thoracic Association (ERS/ESICM/ESCMID/ALAT) define severe pneumonia pragmatically as cases requiring ICU admission. Separately, the American Thoracic Society (ATS) has defined ten criteria for severe CAP, listed in Table 1 [15]. These criteria were all associated with mortality in a multivariate analysis, except hypoxia. The ATS originally defined pneumonia with one major criterion, which was sensitive (98%) but not specific (32%) for predicting mortality, and no combination of criteria could be found that accurately predicted the outcome. Hence, a unifying definition in the literature remains lacking.

### 1.2. Methodology and Literature Review

In order to ensure the breadth of the literature was captured for this review, we employed the Cochrane Handbook for Systematic Reviews search filters, following the ‘Highly Sensitive Search Strategy for identifying randomised trials in MEDLINE’ [16]. We searched the MEDLINE database for any references containing the keywords ‘ICU’, ‘mechanical ventilation’, or ‘critical care’. Publications from this search were then filtered for those containing the keyword ‘bronchoscopy’ and either ‘infection’ or ‘pneumonia’. We considered these terms sufficiently broad to capture the majority of the existing comparative clinical literature, facilitating rigorous scrutiny of contemporary evidence. Despite this relatively non-selective search, only 115 results were identified, which were then manually curated. This further highlights the paucity of systematic evidence guiding the use of this important intervention.

The most widely used guidelines relevant to international practice were selected for discussion. These were often published as collaborative documents involving multiple large multinational societies, for example, the ERS/ESICM/ESCMID/ALAT guidelines described above. Other major guidelines from societies such as the ATS and British Thoracic Society (BTS) were also considered. Regrettably, we could not identify guidelines from many low and middle-income countries, and publications identified in this review were largely from centres in the developed world. This remains a crucial area of unmet clinical need and underscores the importance of supporting clinical research in low-resource settings where the aetiologies of pneumonia may differ significantly.

### 1.3. Development of Bronchoscopy in Intensive Care

Bronchoscopy has provided a valuable window into the respiratory system since its conception over 150 years ago. It has a wide range of diagnostic and therapeutic indications and its scope has increased alongside technological advances during this time. The first bronchoscopes were rigid; fibreoptic bronchoscopy (FOB) was developed in 1968 and revolutionised the field with its comparative versatility, portability, and reduced risk of cross-infection. The ability conferred by FOB to access the distal airways and lung parenchyma with only local anaesthesia and/or mild sedation makes it an attractive bedside choice. With advances in optics and their miniaturisation, rigid bronchoscopy is nowadays generally limited to highly specialised therapeutic applications. FOB has been useful in the ICU for over 50 years [17].

Single-use disposable bronchoscopes are becoming increasingly available, accelerated by the COVID-19 pandemic, making bronchoscopy an ever more accessible diagnostic and therapeutic tool. Indications for FOB in the ICU include localisation and management of haemorrhage, assisting definitive airway placement, clearance of respiratory secretions, and perhaps most commonly, targeted distal airway sampling for infection. Here, we summarise the relevant anatomy and practice of fibreoptic bronchoscopy in the ICU, followed by a discussion of the current evidence for its use as a diagnostic tool in primary and secondary pulmonary infections, including in specific clinical contexts. Finally, we make recommendations for clinical practice and highlight areas requiring further study.

## 2. Relevant Anatomy

The respiratory system exhibits a complex branching architecture adapted for optimal air flow and gas exchange. The lower respiratory tract divides sequentially to form a tracheobronchial tree, mirrored by blood and lymphatic vessels, around which the lung parenchyma is centred. This branching commences with the bifurcation of the trachea to give the right and left main bronchi, and continues until 23 generations have been established.

Airways diverge spatially through the lung parenchyma, their numbers roughly doubling at each generation. The conducting systems (generations 0–16) comprise named macroanatomical structures such as the trachea, bronchi, and bronchioles, and do not have any capacity for gas exchange. They deliver inspired gas to the generations 17–23, comprising the respiratory bronchioles, alveolar ducts, and terminal alveoli, where gas exchange occurs. The airways are lined by pseudostratified ciliated columnar or cuboidal epithelium until the respiratory bronchioles, at which point the epithelium becomes squamous to facilitate gas exchange [18].

**Figure 1:**
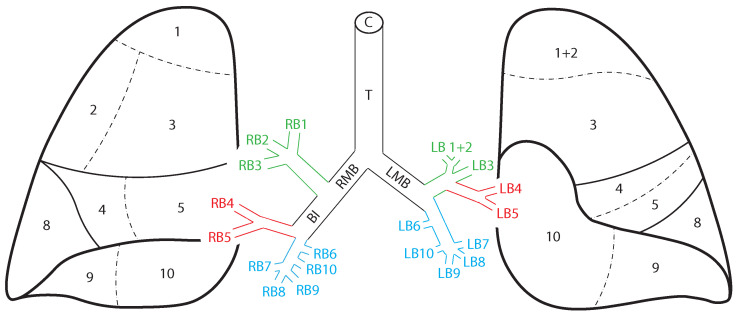
**Anatomy of the human lung.** The surface anatomy of the human lung divided into bronchopulmonary segments is shown here, viewed from the front. Numbered segments correspond to the airway shown in the bronchial tree (i.e., the right upper lobe apical segment 1 corresponds to its segmental bronchus, RB1). The right upper lobe bronchus is divided into three segmental bronchi (RB1–3), the right middle lobe bronchus into two segmental bronchi (RB4–5), and the right lower lobe bronchus into five segmental bronchi (RB6–10). The left upper lobe bronchus gives rise to a fused apicoposterior segment (LB1+2) and an anterior segment (LB3). The lingula bronchus is divided into two segmental bronchi (LB4–5), and the left lower lobe bronchus into five segmental bronchi (LB6–10). In the surface anatomy diagram, the apical (6) and medial (7) segments of the lower lobes are not seen as they are posterior. Solid lines indicate major fissures, while dotted lines indicate non-fissural borders between segments. Green segments show the upper lobes, red segments show the middle/lingula lobes and blue segments show the lower lobes. C, cricoid cartilage; T, trachea; RMB, right main bronchus; LMB, left main bronchus; BI, bronchus intermedius.

**Figure 2:**
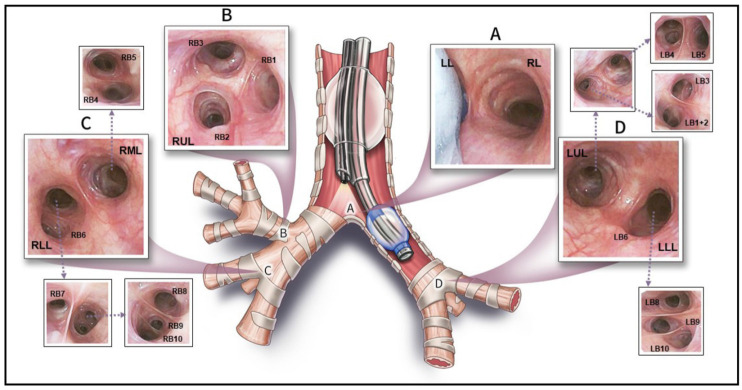
**Major carinae of the human bronchial tree.** Representative pictures of carinae from the human bronchial tree as seen during selective endobronchial intubation. (**A**) The main carina with the right (RL) and left (LL) main bronchi seen during left main stem intubation. (**B**) Canonical right upper lobe anatomy, with segments RB1-3 shown. (**C**) View from the right bronchus intermedius showing the common right lower lobe (RLL) and right middle lobe (RML) bronchi. Arrows show segmental carinae for segments RB4-10. (**D**) View from the left main bronchus, showing the left upper lobe (LUL) and left lower lobe (LLL) bronchi. Above is the view from the LUL bronchus, leading to the upper lobe proper (LB1+2 and LB3) and lingula (LB4–5). Below this are the LLL basal segments (LB8–10). LB6 is seen at the left main carina, and LB7 is not shown. This figure was adapted from Liang et al. [20] under a creative commons CC BY-NC-ND 4.0 licence (https://creativecommons.org/licenses/by-nc-nd/4.0/).

The typical lobar and bronchial anatomy of the human lung is shown in Figure 1. The right lung is divided into upper, middle, and lower lobes; the left into upper and lower principal lobes, with a small lingula lobe which arises from the left upper lobe bronchus. Each lobe is itself divided into wedge-shaped bronchopulmonary segments, each with its own bronchus, arterial supply, and venous and lymphatic drainage. The nerve supply of the lungs arises from the pulmonary plexi posterior to the hila, themselves derived from vagal fibres and 2nd–4th sympathetic trunk ganglia. Most fibreoptic bronchoscopes are of a calibre which allows airway visualisation to the level of the segmental and subsegmental bronchi, whereupon airway diameter limits further scope progression [7,19]. The visual anatomy of the main and principal segmental carinae is shown in Figure 2.

## 3. Alveolar and Bronchial Cell Types in Health and Disease

The anatomical complexity of the respiratory system is reflected in its cellular composition. The respiratory epithelium is specified from the ventral foregut endoderm and lines the developing respiratory tract continuously as it undergoes branching morphogenesis. The mature airways consist of a pseudostratified epithelium supported by a basal lamina of smooth muscle, cartilage, and fibroblasts derived from mesoderm cells; the overall pattern of cell types and their numbers within this framework varies over the proximal-distal axis [21].

Over 40 cell types have been identified in the lungs; the Human Cell Atlas Consortium further characterises these with high spatial resolution across a range of biological contexts, aided by high-throughput single-cell genomic and transcriptomic analyses [22]. The major cell type categories are epithelial, endothelial, stromal, and immune cells [23]. Single-cell RNA-seq and differential cell analyses of samples from the upper airways, lower airways, and parenchyma demonstrate that healthy upper airways contain relatively few immune cells. Conversely, the lower airways are more densely populated by immune cells, which are mostly alveolar macrophages [24].

In the context of bronchoscopic sampling, the cellular composition of bronchoalveolar lavage (BAL) fluid (BALF) is clinically useful. The most abundant cells in BALF are immune cells from the alveolar space and analysing their relative abundances aids the diagnosis of various diseases [25]. Over 80% of the cells isolated from BALF from healthy individuals are alveolar macrophages; around 5–15% are lymphocytes; and neutrophils, eosinophils, and mast cells are present in small numbers [26]. Neutrophils predominate in acute inflammation, and whilst sensitive to infectious causes of pneumonia, they are non-specific and cannot reliably differentiate from non-sterile causes of inflammation and ARDS [27]. In conditions with a known cellular aetiology such as eosinophilic pneumonia, lavage cells may be highly specific. Similarly, a preponderance of lymphocytes progressive interstitial diseases may point to specific diagnoses; however, the findings in many disease states overlap with others and are non-specific [7,28]. Thus, although BALF cell composition may be suggestive of the nature of pulmonary inflammation, it is unable to differentiate between specific aetiologies.

## 4. Bronchoscopic Sampling Techniques

### 4.1. Bronchoalveolar Lavage and Washings

Bronchoalveolar lavage is a technique for sampling the distal alveolar space that is otherwise not accessible using conventional fibreoptic bronchoscopes. Lavage is performed by first wedging the bronchoscope in the appropriate sub-segmental bronchus, then instilling fluid (usually warmed 0.9% saline) via the working channel to form a continuous column from the bronchoscope to the alveolar space. The fluid is then aspirated, with the initial (bronchial) aliquot discarded and cellular (alveolar) fluid collected. Although practice varies, the consensus is that at least 100 mL is required to reliably reach the alveolar space, and a volume of 100–150 mL is commonly recommended, with consensus statements available to guide optimal bronchoalveolar lavage technique in severe acute respiratory failure [19,29]. This allows for sampling of at least $1\times 10^{6}$ alveoli [30].

Bronchial washing involves the instillation of small volumes (20–40 mL) of saline via a bronchoscope without wedging. This technique samples the proximal bronchioles and is more subject to contamination by respiratory flora, which may not reflect the causal pathogen in pneumonia. While this technique does not allow true sampling of the alveolar space, it is a relatively rapid procedure which requires minimal operator expertise in comparison to a true directed BAL which requires careful manipulation of the bronchoscope to an anatomically defined location and often hand-driven aspiration to optimise alveolar fluid returns.

### 4.2. Protected Specimen Brush

Protected specimen brushes (PSB) are sterile brushes that are advanced down the working channel of the bronchoscope and allow sampling of the airways distal to the scope, minimising contamination from the trachea and proximal airways. The brush does not extend much beyond the end of the scope and therefore samples the respiratory surface of the proximal bronchioles. Whilst this shows similar microbiological features to more distal samples from lavage [31], the cellular components are different and reflect the more proximal nature of the sample. Thus, BAL may have greater diagnostic utility in cases of uncertainty as to the aetiology of alveolar infiltrates.

### 4.3. Blind Bronchial Sampling Techniques

Non-directed mini bronchoalveolar lavage (mini-BAL) was first described in 1987 by Mann and colleagues [32]. It is a blind technique which involves endotracheal advancement of a lavage catheter, instillation of small volumes of saline, and subsequent aspiration to obtain the sample. This may be performed using a bespoke double-lumen lavage catheter as originally described, or by using widely available flexible suction catheters after blind endotracheal instillation of saline flushes [33]. Mini-BAL is a safe, rapid, reproducible, and cost-effective technique which requires minimal operator experience and relies on widely available materials and is effective in diagnosing VAP [34]. However, as it does not sample the true alveolar space, it may be ineffective in the diagnosis of anatomically localised VAP and is susceptible to contamination by commensal airway organisms.

### 4.4. Direct Bronchial Examination and Novel Optical Techniques for Alveolar Visualisation

Given the drawbacks of current practice in diagnosing VAP, there is a clinical need for tests to more accurately and quickly identify or exclude the presence of a causative organism [35]. One of the advantages inherent to bronchoscopy is the ability it gives clinicians to perform a visual inspection of mucosal surfaces. For example, *Aspergillus* tracheitis has a characteristic macroscopic appearance; the presence of pus within a segment or subsegment of a lung has a high positive predictive value for bacterial pneumonia. Similarly, in cases of bronchial obstruction and lung collapse, endobronchial infection mimics such as obstructing lung tumours can be rapidly identified, biopsied, and confirmed by histopathological examination. However, the resolution of information that can be derived from crude visualisation alone is limited. In recent years, novel optical bronchoscopic technologies for visualising the alveoli have proven promising in addressing these issues. In 2018, Dhaliwal and colleagues developed an imaging method for the detection of Gram-negative bacteria in the distal lung in real time. A fluorophore-conjugated polymyxin probe binds to lipid A on Gram-negative bacterial membranes which are subsequently visualised with optical endomicroscopy [36].

The Translational Healthcare Technologies group has recently developed an optical molecular alveoscopy (OMA) platform that facilitates bedside diagnosis in the ICU. In this method, fluorescent molecular imaging probes directed against important elements of pneumonia (e.g., bacteria, activated neutrophils) are delivered into the distal lung via the working channel of a bronchoscope [35]. Another platform, termed fibered confocal fluorescence microscopy (FCFM), can detect matrix metalloprotease (MMP) activity using a bronchoscope-compatible delivery device. MMP activity is implicated in numerous inflammatory respiratory diseases [37]. Detection of host enzyme activity in the lungs may facilitate better differentiation between sterile and infective causes of respiratory failure and a more personalised impression of a patient’s disease process. These technologies are currently undergoing clinical evaluation before being made more widely available in the future.

## 5. Molecular Microbiology

This growing arsenal of optical tools is complemented by advances in molecular diagnostics. In the conventional diagnostic pipeline, BALF culture and sensitivities incur an obligate delay of up to 72 h. However, an expanding array of rapid polymerase chain reaction (PCR) based molecular diagnostics is increasingly mitigating this critical drawback of conventional practice. Many ICUs globally have access to molecular diagnostics and their availability is likely to increase further [6]. This shifts the risk–benefit balance of bronchoscopy and should prompt a reassessment of its merits in this new context.

In recent years, rapid syndromic tests for bacterial pneumonia have become commercially available [38] and demonstrated great promise. In the INHALE trial, 652 respiratory samples were analysed using conventional microbiology and two multiplex PCR platforms. Both platforms were considerably more sensitive than routine microbiology [39]. The development of a 52-respiratory-pathogen TAQman array card (TAC) allows the rapid and highly sensitive detection of multiple pathogens and alters prescribing. The TAC is also customisable and amenable to rapid modifications so can be adapted to emerging threats [40]. Its utility extends to critically ill children and may facilitate early rationalisation of antimicrobials in the paediatric ICU [41].

The inclusion of viral panels in diagnostic tests further aids differentiation between causes of ARF and reduces antibiotic overuse [42]. Further enhancing their clinical value, several syndromic tests now include assays for key antimicrobial resistance (AMR) genes, including MecA, which induces methicillin resistance, and NDM-1 and blaKPC, which encode key carbapenemases [42]. There is mounting evidence that metagenomics may take on an increasing role in diagnostics in critical care. A recently developed 6 h nanopore sequencing respiratory metagenomics workflow demonstrated great potential in ventilated patients with coronavirus disease (COVID-19). This workflow influenced prescribing decisions in 80% of cases and prompted early immunomodulation for suspected inflammatory lung conditions where infection was excluded [43].

False negative results from molecular tests can have adverse consequences, and a decision to withhold antibiotics in the absence of detected organism needs to be considered in the context of the range of organisms on the test and the pre-test probability of bacterial pneumonia based on the patient presentation [42]. However, this must be balanced with the risk of mistaking detectable colonisation for infection. A combination of highly sensitive molecular diagnostics and protected lower respiratory tract samples that are less likely to detect colonising organisms may represent the optimal balance between sensitivity and specificity. However, the impact on patient outcomes is currently uncertain and the merits of proximal vs. distal sampling in the context of molecular diagnostics remain to be elucidated.

## 6. Safety of Bronchoscopy

**Figure 3:**
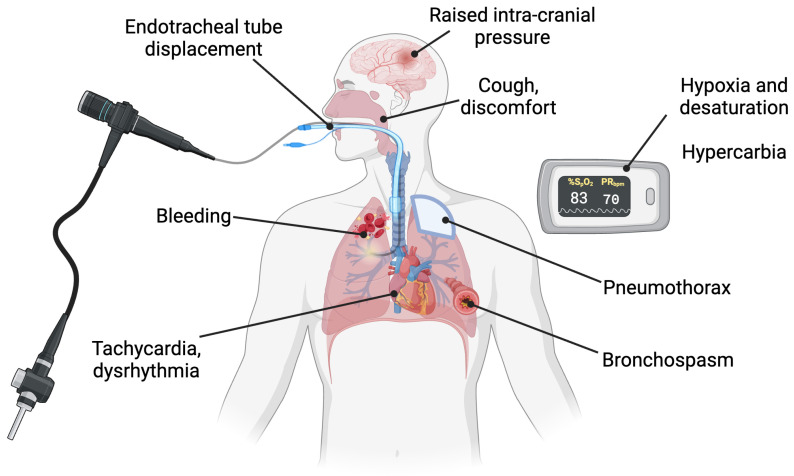
**Common complications of ICU bronchoscopy.** Categories of complications of bronchoscopy include cardiovascular (bleeding, bradycardia, hypotension); respiratory (hypercapnia, hypoxia, bronchospasm, pneumothorax); and symptomatic (pain, breathlessness, cough). These can be mitigated by measures frequently used in the ICU, such as sedation. Importantly for ICU bronchoscopy, meticulous attention must be given to endotracheal tube security to prevent displacement.

Although semi-invasive in nature, bronchoscopy is widely regarded as safe. Its mortality rate is reported as 0.01–0.04% and its complication rate below 0.3% [19], although this is higher in intensive care settings, where complication rates of up to 10% are reported [44]. The effects on the physiology of the respiratory system—increased airway resistance; reduced lung compliance; hypoxaemia and hypercapnia; and cardiovascular effects—explain the risks of the procedure [19]. Common complications are illustrated in Figure 3.

Hypoxaemia, haemorrhage, and pneumothorax constitute the major risks of bronchoscopy. Hypoxaemia is caused by increased airway resistance, in turn caused by occlusion of a fraction of the trachea by the bronchoscope and potentially endotracheal tube, and alveolar flooding [19]. Appropriate bronchoscope selection can help mitigate this, with a recommendation that the external diameter for the scope should be at least 2 mm smaller than the internal diameter of the endotracheal tube [45]. Additional strategies to avoid hypoxaemia include pre-oxygenation, positioning the patient head-up, and the use of neuromuscular blockade. Airway pressures rise during the procedure, posing the risk of pneumothorax, which can be mitigated by careful monitoring of ventilator settings and efficient technique [7].

Reduced lung compliance is likely caused by distal airway collapse secondary to the effects of suction and saline lavage and resulting changes in surfactant. Hypercapnia is underpinned by hypoventilation due to airway obstruction. Effects on heart rate and blood pressure stem from sympathetic stimulation and the effects of sedatives. Hypotension requiring vasopressors occurs in around 22% of patients in the ICU and can be exacerbated during bronchoscopy. However, high standards of clinician training, careful patient selection, knowledge of the risks, and appropriate mitigation measures allow the procedure to be conducted safely in ventilated, critically ill patients [46]. Concerningly, confidence in bronchoscopy and reported formal training amongst intensivists is highly variable [6,47]. Reported practice in lavage volumes is also highly variable and seldom conforms with consensus standards for bronchoscopy [6]. We suggest this requires urgent attention from training and regulatory bodies worldwide.

## 7. Evidence for Bronchoscopic Sampling in Severe Respiratory Infections

### 7.1. Timing of Bronchoscopy

Ideally, sampling for causative agents in the context of bacterial infections should occur prior to the administration of antibiotics. However, an association between antibiotic timing and mortality constitutes an important caveat; sampling should not delay timely medication [48,49]. For less invasive, technically simpler procedures such as blood cultures, balancing early pre-antibiotic sampling and timely medication is a realistic goal. However, for a specialist technique such as bronchoscopy, appropriately trained clinicians may not be present when infection is identified [6]. Thus, empirical therapy is frequently given prior to bronchoscopy, which may limit the sensitivity of conventional culture techniques.

In a study involving 63 cases of VAP, recent antibiotic administration reduced the sensitivity of BAL from 78% to 38%, highlighting the effects of even short antibiotic courses [50]. Additionally, in a separate study, three days of de novo antibiotic therapy was capable of complete eradication of susceptible organisms in 94% of samples from patients with VAP [51]. These data suggest that earlier timing of bronchoscopy may improve culture yield and organism identification in patients with bacterial aetiologies. This advantage must be balanced with the importance of early antibiotic administration and the availability of trained personnel. The use of molecular diagnostics, discussed below, may help reduce this dilemma.

### 7.2. Ventilator-Associated Pneumonia

VAP has an incidence of up to 35% among critically ill patients mechanically ventilated for 48 h and carries a mortality rate of up to 27% [19]. Diagnostic practices for VAP vary significantly globally [6,52]. The bulk of the available literature regarding bronchoscopy for diagnosing respiratory infections is in the context of VAP. Invasive sampling may seem intuitive; as BAL captures cells and organisms from the alveolar compartment, it is singular in its ability to confirm or refute the presence of infection at this anatomical level. A single-centre prospective study of BAL for the diagnosis of VAP in a post-surgical population demonstrated an overall sensitivity of 80% and specificity of 66% when compared to the gold standard of histopathologic examination [53]. Subsequent work from this team demonstrated similar diagnostic performance in another cohort [54]. Additionally, an alveolar neutrophil percentage of <50% in BALF combined with a negative Gram stain can exclude bacterial pneumonia with a negative predictive value of close to 100% [7]. However, invasive techniques are likely only beneficial if they alter practices such as antibiotic prescribing; otherwise, they are unlikely to alter outcomes such as ICU length of stay or mortality.

In 2000, Fagon et al. found in a randomised controlled trial that invasive sampling was associated with lower 14-day mortality when antibiotics were held following a negative result on bronchoscopy [55]. However, several subsequent studies demonstrated that negative respiratory cultures alone were insufficient to drive clinicians to discontinue antibiotics, making it less likely that tangible clinical outcomes be affected by bronchoscopy [7,56,57,58]. A 2006 multi-centre randomised trial comparing ETA and BAL for VAP diagnosis did not reveal any significant differences between the groups in 28-day mortality, ICU length of stay, or organ dysfunction [59]. More patients in the BAL than the ETA group (59.7% vs. 51.9%) had a positive culture. However, patients were treated empirically with broad-spectrum antibiotics, and therapy was continued in some cases of low pre-test probability of VAP and negative culture [59]. Observational studies suggest that discontinuation of antibiotics in VAP can be carried out safely and results in fewer multi-drug resistant superinfections [60].

Fernando et al. concluded in a 2020 systematic review and meta-analysis of 25 studies and 1639 patients that classic clinical indicators including fever, purulent secretions, leukocytosis, chest radiography, ETA, and bronchoscopic samples all have low specificity for diagnosing VAP. However, of these, bronchoscopy has the highest specificity at 79.6%. The authors note that pooled estimates are of low certainty owing to poor study quality, and frequent lack of histopathological gold standard [31]. Importantly, studies often do not disclose whether antibiotics were administered prior to bronchoscopy; this will affect the reported sensitivity. Targeted antibiotic therapy (discontinued or adjusted antibiotics) based on the results of bronchoscopy has been shown to be safe and associated with improved clinical outcomes [61]. A recent large retrospective study found that bronchoscopy was associated with significantly reduced ICU and in-hospital mortality [62]. However, the antibiotic treatment of these patients before and after bronchoscopy was not evaluated. Another study showed that ETA and BAL culture results were concordant in only 53% of patients with VAP diagnosed bronchoscopically [63]. A switch from an endotracheal aspirate to a bronchoscopic diagnostic approach in suspected VAP was associated with a reduction in the microbiological diagnosis of VAP, antibiotic use and mortality [64].

Bronchoscopy and BAL may also be useful in diagnosing early pneumonia in critically ill trauma patients, allowing delineation between traumatic lung injury and VAP and preventing spurious diagnosis of the latter [65]. Combinatorial diagnostic algorithms incorporating early BALF parameters and clinical and radiological data may outperform the sensitivity and specificity of any variable alone [66].

Overall, the evidence for bronchoscopy in the diagnosis of VAP is not strongly compelling. However, this is likely largely attributable to antibiotic prescribing practices and low-quality studies. A recurring caveat to otherwise high-quality studies is that negative results on bronchoscopy often fail to translate to modified antibiotic therapy. A recent randomised controlled trial (VAPrapid2) across 24 UK ICUs reconfirmed this pattern: despite BALF biomarker-informed recommendations to discontinue antibiotics, antibiotic use was not changed and no significant changes in clinical outcome were observed [67].

Where antibiotics are targeted following bronchoscopy, bronchoscopy is associated with improved clinical outcomes [55,62]. In light of this, a review article published in 2022 concluded that, all things considered, invasive sampling is favourable [7]. High-quality randomised trials would clarify the extent of the clinical utility of bronchoscopy in VAP diagnosis. Additionally, standardised clinical guidelines regarding antibiotic usage following bronchoscopy would allow this knowledge to translate to improved outcomes.

### 7.3. Immunocompromised Patients in the ICU

Immunocompromised patients are especially at risk of developing severe respiratory infections, which are associated with high mortality in this group. This population of patients includes those with prolonged neutropaenia, allogenic haematopoietic stem cell or solid organ transplant recipients, those with inherited or acquired immunodeficiencies, and those who have been treated with high-dose or prolonged corticosteroids [68]. A multi-centre study of 1611 immunocompromised patients admitted to the ICU for acute respiratory failure found that fungal infections were responsible for 14% of these [69,70]. Due to the scale of the clinical problem presented by invasive fungal respiratory infections, along with their increasing incidence in immunocompetent patients [71], they will be discussed in a separate section.

In a general non-HIV immunocompromised critically ill population, a 2018 prospective observational study concluded that flexible bronchoscopy is safe and its yield is improved when performed prior to empirical antibiotic administration [72]. The impact of bronchoscopy on management was most notable in patients receiving corticosteroids and those who had recently received chemotherapy, and lowest in patients receiving non-corticosteroid immunosuppressive therapy. Focal, rather than interstitial or diffuse, radiological findings are predictive of higher diagnostic yield. Authors suggest that bronchoscopy be performed within 24 h of ICU admission due to improved diagnostic yield in this window [72].

Among high-risk immunocompromised patients with haematological malignancies, pulmonary complications are a significant source of morbidity and mortality. Around 85% of patients undergoing antileukaemic chemotherapy develop infections and/or fever [73]. However, pulmonary infiltrates may also be caused by haemorrhage, neoplasia, or treatment-related toxicity. In acute respiratory failure (ARF), early identification of pulmonary infiltrate aetiology is associated with better outcomes [74], and is crucial in the face of emerging aetiologies of sterile infiltration in this population, notably from checkpoint inhibition [75].

Bronchoscopy is safe and valuable in the diagnosis of respiratory infections in this group; its diagnostic yield was 47.9% and its utilisation led to antimicrobial modification in 38.2% of cases [73]. An earlier study reported an overall diagnostic yield of 49% and a higher yield in those with chemotherapy-induced neutropaenia [76]. Within the umbrella of patients with haematological malignancies admitted to the ICU for ARF, bronchoscopy has a lower diagnostic yield in acute myeloid leukaemia (AML) than lymphoid malignancies including non-Hodgkin’s lymphoma, Hodgkin’s lymphoma, chronic lymphocytic leukaemia, and multiple myeloma [74]. In a retrospective study of febrile neutropaenic patients with lung infiltrates, 85.6% of whom had haematological malignancies and the remaining had solid organ malignancies, bronchoscopy was safe and useful. It exhibited sufficient diagnostic yield to alter management in the majority [77].

Similarly, bronchoscopic sampling led to altered antibiotic prescription in 35% of critically unwell lung transplant recipients [78]. Furthermore, bronchoscopy was the best investigation for diagnosing non-ventilator ICU-acquired pneumonia following cardiothoracic surgery and preventing excessive antibiotic treatment [79].

High-quality randomised controlled trials examining the merits of bronchoscopy among immunocompromised patients in the ICU are lacking. However, the available evidence suggests that bronchoscopy is a safe means of aiding the diagnosis of respiratory infections and affects antimicrobial prescribing across a wide range of aetiologies of immune dysfunction. As in VAP, empirical antibiotics likely affect the diagnostic yield.

### 7.4. Invasive Fungal Infections

Among immunocompromised patients, fungal infections constitute a significant threat. However, invasive fungal diseases can also present in previously immunocompetent patients without the associated classical host factors. The immune dysfunction that occurs paradoxically in concert with hyperinflammation in sepsis is implicated in the higher risk of contracting opportunistic infections that critically ill patients face [80]. Furthermore, the medications and clinical procedures patients are exposed to in the ICU may affect their susceptibility to fungal infections [71,81,82].

Invasive fungal infections of the respiratory system carry very high mortality of >80% if not met with sufficiently aggressive treatment [19], partially attributable to non-specific signs and symptoms and their resulting under-diagnosis in the ICU [69]. As such, re-evaluating the merits of invasive respiratory sampling in this context is worthwhile. Infections with *Aspergillus* species constitute the most commonly encountered invasive respiratory fungal infections in the critical care setting and are therefore the focus of this section. Mucormycyosis is rare but growing in prevalence. *Candida* species are commonly isolated from the lungs of critically ill patients but are seldom thought to cause pneumonia. They may, however, represent heavy candidal colonisation, a known risk factor for candidaemia.

The lungs are the most common site of *Aspergillus* infection, and *Aspergillus fumigatus* is the most frequent causative species [69]. The incidence of invasive aspergillosis (IA) in the ICU is up to 5.8%, although this varies globally. BAL cultures have a sensitivity of around 50%, with histopathological examination remaining the gold standard for diagnosis, despite its invasive nature and the resulting high threshold for carrying it out in this vulnerable population [69].

Differentiating IA from colonisation can be a challenge, so clinical reasoning should encompass clinical signs and patient risk factors. The polysaccharide galactomannan (GM) is a a fungal cell wall component that may be quantified by enzyme-linked immunosorbent assay (ELISA) on serum or BALF. The latter has a reported sensitivity of up to 86%, specificity of up to 91%, and positive and negative predictive values of 80% and 95%, respectively. However, among other factors, beta-lactam antibiotic medications may result in false positives [69]. Lateral flow test devices that sample serum or BALF have become available, enabling results within 1 h.

A recent meta-analysis found no difference between the accuracy of lateral flow assay (LFA) alone and a combination of GM and LFA in diagnosing IA; LFA is advisable if timely results are of the essence [83]. A prospective study of critically ill non-neutropaenic patients found that bronchoscopy is a reliable procedure with high sensitivity and specificity for the diagnosis of tracheobronchial aspergillosis (TBA), a form of IA limited to the tracheobronchial tree, in this cohort. However, this applies to macroscopic assessment on bronchoscopy in addition to BALF analysis [84].

The COVID-19 pandemic accelerated investigations into the merits of bronchoscopy in diagnosing fungal infections because IA emerged as a common complication among critically ill COVID-19 patients. COVID-19-associated pulmonary aspergillosis (CAPA) is discussed in detail in the following section, and the insights these studies yielded are applicable to non-COVID-19-associated IA.

### 7.5. COVID-19

International recommendations regarding the use of bronchoscopy were issued from expert panels and scientific societies in 2020; however, technical guidance was vague and clinical practice was heterogeneous [85,86,87,88]. Bronchoscopy appears safe and of both therapeutic and diagnostic value in patients with SARS-CoV-2 pneumonia [89,90]. In patients with clinical and radiological signs but negative nasopharyngeal swab results, it aids differentiation between COVID-19 and its mimics [88], and is also useful in detecting bacterial superinfections. Targeted antibiotic therapy can then be initiated to prevent over-treatment with broad-spectrum antibiotics. However, concerns regarding aerosol generation and risk of transmission limited the use of bronchoscopy during the pandemic [91], despite published strategies for mitigating these risks [92].

As the COVID-19 pandemic evolved, it became apparent that CAPA is a common complication of COVID-19 among critically ill patients and confers very high mortality [93]. Respiratory viruses damage the respiratory epithelium, permitting fungal invasion [91]. The incidence of CAPA among critically unwell COVID-19 patients was roughly 2% in one large prospective observational study [91]. As such, timely diagnosis of CAPA is important. BALF GM testing had a high sensitivity (84.9%) and BALF metagenomic next-generation sequencing facilitated earlier diagnosis, leading to authors recommending timely bronchoscopy in patients with suspected CAPA [94]. ETA was shown to be inferior to BAL, albeit with a high negative predictive value, so is recommended where bronchoscopy is not possible [95].

Testing BALF with an *Aspergillus* lateral flow device (AspLFD) is reasonably sensitive and highly specific for CAPA, also enabling rapid diagnosis [93]. Metagenomic and metatranscriptomic analyses of the lower respiratory microbiome and host immune profiling were conducted on BALF from critically ill mechanically ventilated COVID-19 patients. SARS-CoV-2 abundance was identified as a predictor of mortality, along with certain host transcriptomic signatures and immune cell abundances. Authors were able to discern that poor specific antibody responses and SARS-CoV-2 replication, rather than secondary infections, drove increased mortality [96].

The role of the lung microbiota in severe COVID-19 infections was further clarified in 2022; increased bacterial and fungal burden on BAL correlated with lower probability of extubation and higher mortality [97]. Bronchoscopy is also useful in diagnosing viral superinfections, revealing high rates of HSV and CMV reactivation in the lung; however, these were not associated with altered outcomes [98]. One study found that mini-BAL is a useful tool for screening for CAPA, despite not being as effective as BAL for diagnosis [99].

### 7.6. Bronchoscopy in Critically Ill Paediatric Patients

The literature concerning bronchoscopy in paediatric populations is more sparse compared to that for adults. However, one 2008 UK study concluded that flexible bronchoscopy “should be seen as a routine diagnostic and therapeutic tool in paediatric intensive care” [46]. This is reiterated in later studies. One 2023 paper studying bronchoscopy in 229 children in a Chinese paediatric ICU found that early BAL reduced the duration of ICU stay but not mortality; the authors plan to conduct a larger multi-centre trial to further investigate the role of bronchoscopy in this population [100]. Among neonates, bronchoscopy has diagnostic and therapeutic value and affects antimicrobial prescription [101]. In a resource-limited setting, authors concluded from a small prospective study that non-bronchoscopic blind BAL in children in the paediatric ICU is most appropriate for diagnosing VAP [102].

## 8. Bronchoscopic Sampling in Differentiating Infective from Sterile Inflammatory Disease

Identifying and treating the underlying cause of critical illness is vital. However, diagnosis in the ICU is often imprecise despite complex pathophysiology and marked individual variation in the way patients react to critical illness of both infectious and sterile aetiologies. In the context of the respiratory system, ARDS is often used as a diagnosis but is comprised of a collection of diseases which necessitate specific treatment. Although infective and sterile causes of ARDS warrant distinct treatment paradigms, the clinical and immunological responses they elicit from the host exhibit significant overlap due to converging molecular pathways centred on pattern recognition receptors (PRRs). Profiling the host immune response to inflammation promises to aid differentiation between sterile and infective inflammation and disentangle the latter from colonisation [5]. A variety of means of transcriptional, proteomic, and functional profiling are available [5]. These methods may help bring a precision medicine revolution to the ICU.

Exemplifying the importance of delineating sterile inflammatory lung injury from infection is that non-infective exacerbations of interstitial lung disease (ILD) mimic ARDS, where disease modification by immunosuppressive therapies is necessary. Differential cell counts may show lymphocyte-rich BALF, which in conjunction with high-resolution CT scanning may support a diagnosis of ILD subtypes such as sarcoidosis or hypersensitivity pneumonitis [103]. However, similar BALF findings are common in viral pneumonia, so this is an imperfect method for identifying sterile inflammation. Similarly, interstitial eosinophilia is highly suggestive of primary pulmonary eosinophilic disease, but may also occur in parasitic lung infections. Thus, BALF cellularity must be carefully interpreted when used to rule out severe respiratory infection.

## 9. Current Clinical Guidelines

The most recent British Thoracic Society (BTS) guidelines concerning diagnostic flexible bronchoscopy in adults were published in 2013. They state that directed non-invasive diagnostic strategies should be used first line in the diagnosis of VAP, but that when such non-invasive diagnostic techniques fail to identify a responsible organism, bronchoscopy should be considered for the diagnosis of VAP [104]. This advice overlooks that ETA is likely to identify an organism, but it is difficult to distinguish tracheal colonisation from infection. Contraindications to bronchoscopy include active myocardial ischaemia and continuous ECG monitoring is recommended where there is a high clinical risk of arrhythmia [104]. Current guidelines suggest that bronchoscopy with lavage can be performed with platelet counts of over 20,000 per μL. However, platelet transfusion prior to diagnostic bronchoscopy does not reduce bleeding risk in thrombocytopaenic patients, and overall bleeding complications are low even for patients with platelet counts under 20,000 per μL [105]. This suggests this target may be unduly conservative and warrants further systematic study.

The Infectious Diseases Society of America (IDSA) and ATS published guidelines on the management of adults with HAP and VAP in 2016. These guidelines suggest non-invasive sampling (ETA) with semi-quantitative cultures but designate this as a weak recommendation based on low-quality evidence. However, the guidelines also emphasise antibiotic de-escalation to minimise patient harm, exposure to unnecessary antibiotics, and the development of antibiotic resistance. They suggest that antibiotics be held if invasive quantitative cultures are below the diagnostic threshold for VAP. Again, this is designated a weak recommendation and the included remarks highlight the importance of clinical judgement [106]. Overall, the guidelines note that the literature does not reveal a significant difference between outcomes after invasive and non-invasive sampling but fails to question whether this is due to prescribing practices.

The 2017 ERS/ESICM/ESCMID/ALAT guidelines for the management of HAP and VAP [13,14] are the most recently published. They suggest obtaining distal quantitative samples prior to antibiotic treatment to reduce antibiotic exposure in stable patients with suspected VAP. The authors highlight differences between these European/Latin American guidelines and the US guidelines and give reasoning as to why this divergence exists. Firstly, it is acknowledged that definitions of HAP and VAP differ somewhat. Additionally, authors point out that while ventilator-associated complications are widely used as a surrogate measure of VAP in the USA, a lack of sensitivity and specificity has precluded this in Europe [14].

Overall, there is a dearth of evidence-based guidelines regarding bronchoscopic sampling in respiratory infections in the ICU. The guidelines are also at least 7 years old. This is significant as the COVID-19 pandemic saw an increased output of studies evaluating the use of diagnostic bronchoscopy and recognition of CAPA in the ICU. The past decade has also seen significant developments in optical bronchoscopic techniques and molecular diagnostics. Furthermore, despite sharing a common body of literature, the recommendations in the three different guidelines diverge notably. Future directions for the field should include the generation of high-quality studies along with up-to-date guidelines.

## 10. Conclusions, Future Directions, and Recommendations for Clinical Practice

In conclusion, although not universally implemented, diagnostic bronchoscopy is well established in the ICU. It offers clinicians the ability to perform directed, protected sampling for infection, cellular makeup, and haemorrhage at the bedside. It is clear that at the heart of the debate surrounding its application to respiratory infections in the ICU is a risk–benefit calculation based on a trade-off between sensitivity and specificity. Non-invasive sampling is likely to identify organisms, but these may be colonising, and patients may then be exposed to unnecessary broad-spectrum antibiotics. In the case of bronchoscopy, the procedure itself is semi-invasive and confers a further degree of risk to already vulnerable patients. However, high standards of clinical practice allow mitigation of the risk this procedure confers and its appropriate use has the potential to identify the precise aetiology of ARF.

The combination of bronchoscopic sampling and rapid molecular diagnostics may allow simultaneously high specificity and sensitivity. Importantly, this might circumvent the clinical harm of irrational antibiotic use. High-quality studies and up-to-date guidelines are warranted and their generation should be a priority for the field. It is also important that standards for training and practice be agreed upon to maximise the safety of this technique. Currently, evidence suggests that bronchoscopy is of particular benefit in VAP, where there is a high risk of sample contamination, among immunocompromised patients, and in invasive aspergillosis. In all cases, informed by these complexities and their patient’s unique situation, clinicians must use their judgement to weigh up which diagnostic process has the most favourable risk–benefit profile.

## Figures and Tables

**Table 1:** **ATS severity criteria for community-acquired pneumonia.** The ATS define ten criteria that can be used to identify severe community-acquired pneumonia. These are divided into six minor and four major criteria. Three criteria are respiratory (respiratory rate, hypoxia, and mechanical ventilation); three are radiologic (bilateral or multilobar involvement, or radiologic progression); and the remaining four pertain to extrapulmonary organ failure (blood pressure, vasopressor requirement, and renal failure). Adapted from Ewig et al. [15].

Minor Criteria	
Respiratory	Respiratory rate >30 breaths per minute
	Hypoxia (Pa_O_2__/Fi_O_2__ ratio <250 mm Hg
Radiologic	Bilateral pneumonia
	Multilobar involvement
Extrapulmonary	Systolic blood pressure <90 mm Hg
	Diastolic blood pressure <60 mm Hg
**Major Criteria**	
Respiratory	Mechanical ventilation
Radiologic	Increase in infiltrate size by >50% despite treatment
Extrapulmonary	Vasopressor requirement
	New onset renal failure
